# Explainable artificial intelligence (XAI) detects wildfire occurrence in the Mediterranean countries of Southern Europe

**DOI:** 10.1038/s41598-022-20347-9

**Published:** 2022-09-29

**Authors:** Roberto Cilli, Mario Elia, Marina D’Este, Vincenzo Giannico, Nicola Amoroso, Angela Lombardi, Ester Pantaleo, Alfonso Monaco, Giovanni Sanesi, Sabina Tangaro, Roberto Bellotti, Raffaele Lafortezza

**Affiliations:** 1 Dipartimento Interateneo di Fisica M. Merlin, Università degli Studi di Bari Aldo Moro, Bari, Italy; 2 Dipartimento di Scienze Agro-Ambientali e Territoriali (DiSAAT), Università degli Studi di Bari Aldo Moro, Bari, Italy; 3 Dipartimento di Farmacia-Scienze del Farmaco, Università degli Studi di Bari Aldo Moro, Bari, Italy; 4 Istituto Nazionale di Fisica Nucleare, Sezione di Bari, Bari, Italy; 5 Dipartimento di Scienze del Suolo, della Pianta e degli Alimenti, Università degli Studi di Bari Aldo Moro, Bari, Italy; 6 Department of Geography, The University of Hong Kong, Centennial Campus, Pokfulam Road, Pokfulam, Hong Kong China

**Keywords:** Environmental impact, Governance, Climate-change policy

## Abstract

The impacts and threats posed by wildfires are dramatically increasing due to climate change. In recent years, the wildfire community has attempted to estimate wildfire occurrence with machine learning models. However, to fully exploit the potential of these models, it is of paramount importance to make their predictions interpretable and intelligible. This study is a first attempt to provide an eXplainable artificial intelligence (XAI) framework for estimating wildfire occurrence using a Random Forest model with Shapley values for interpretation. Our findings accurately detected regions with a high presence of wildfires (area under the curve 81.3%) and outlined the drivers empowering occurrence, such as the Fire Weather Index and Normalized Difference Vegetation Index. Furthermore, our analysis suggests the presence of anomalous hotspots. In contexts where human and natural spheres constantly intermingle and interact, the XAI framework, suitably integrated into decision support systems, could support forest managers to prevent and mitigate future wildfire disasters and develop strategies for effective fire management, response, recovery, and resilience.

## Introduction

Mounting climate change effects are exacerbating wildfire distribution and occurrence worldwide. The ensuing ecological and social impacts continuously pose new challenges to the implementation and enforcement of effective disaster risk management and preventive measures for wildfire occurrence^1–3^. It is of paramount importance to provide accurate and easily interpretable frameworks to detect wildfire occurrence across large geographical regions such as the Mediterranean countries of Southern Europe.

Wildfire risk is an extremely complex phenomenon to model due to its interdependency with multiple factors whose relationships are difficult to predict. In recent years, several studies have addressed the intrinsic complexity of such an issue with agnostic approaches provided by machine learning (ML)^4–7^. These techniques have proven to be highly accurate and reliable for wildfire risk assessment^5^; yet, there is still reticence and distrust on the part of stakeholders in using ML models because ML is often considered a “black box” whose results are difficult to interpret^8^. A ML model is transparent if it is clear on how it makes its decisions. Justification and informativeness describe a model’s ability to elucidate why a specific decision is acceptable and to provide new knowledge to decision-makers, respectively. In addition, ML model uncertainty should be robustly assessed to precisely quantify the extent to which its decisions are reliable. In the last decade, there has been a growing scientific interest in the development of a particular ML approach, known as eXplainable artificial intelligence (XAI)^9,10^, capable of creating learned models and decisions that are understood and effectively trusted by end-users. Generally speaking, a unanimous definition has not been attributed to XAI^11^; however, it has been demonstrated that XAI explicates relevant aspects, such as transparency, justification and informativeness, which are of crucial importance, especially for social or clinical applications^12–14^. ML models can be extremely complex; a popular example worth mentioning are neural networks. In many cases these models become ``black boxes’’ and it is difficult to extrapolate general rules relating input features and output scores. In this perspective, any approach that attempts to develop an intelligible a model may fall within the XAI domain.

In light of the above, we present a first attempt to elaborate an XAI framework for wildfire occurrence assessment over the entire Italian peninsula by using European Space Agency (ESA) Corine Land Cover maps, Open Street Map (OSM) data, climate data and the Normalized Difference Vegetation Index (NDVI). We evaluate the extent to which the informative content provided by these data allows a reliable and accurate prediction of wildfire occurrence. By confirming the findings from previous studies^15–17^, we demonstrate that such a knowledge base can accurately predict wildfire occurrence. Therefore, it is reasonable to adopt an XAI framework to identify driving factors and their interactions. Furthermore, based on analyses of the spatial distribution of wildfires in Italy, we outline the presence of anomalous wildfire hotspots, i.e., regions with a significantly anomalous presence of wildfire spatial clusters.

## Materials

Our analyses focused on the Italian peninsula which lies at the center of the Mediterranean Basin. The Italian territory extends over about 300,000 km^2^ and is roughly divided into lowland (23%), upland (42%) and mountain (35%) regions. The peninsula basically follows a North-South climate gradient, affecting the wildfire regime. In Northern regions wildfires are most frequent from January to March, whereas in Southern regions the wildfire season is mostly concentrated in the summer months when dry winds from North Africa create conditions for extreme events.

**Figure 1:**
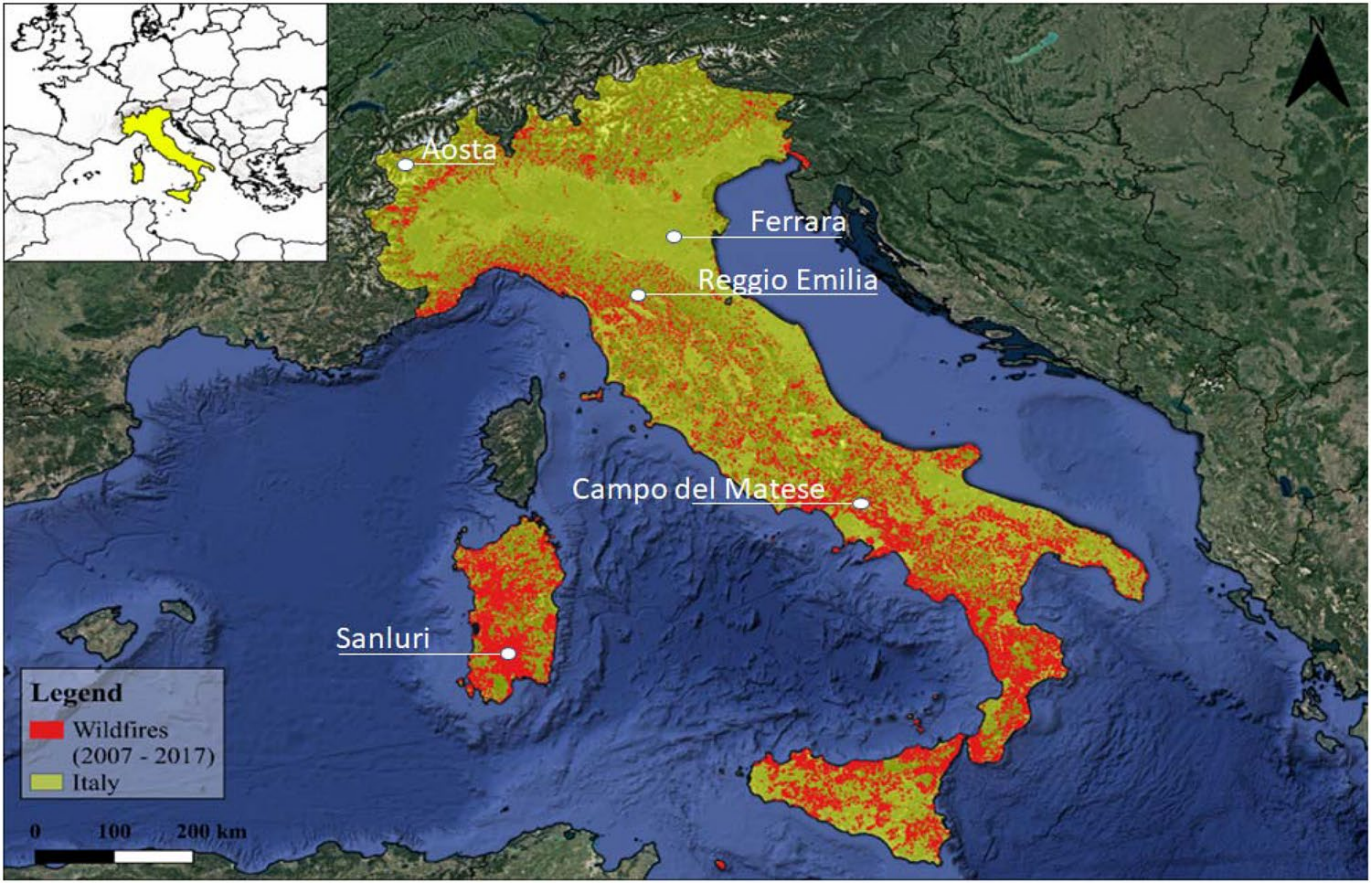
Map of the Italian peninsula (yellow) in the Mediterranean basin illustrating the distribution of wildfire occurrences (red) across the period of investigation. The map was produced on QGIS using the "new layout" feature.

In the present study, a shapefile dataset of fire polygons (fire perimeters) was processed to create a binary occurrence map to be used as a response variable in the subsequent analyses. The wildfire dataset was provided by the Comando Unità Forestali, Ambientali e Agroalimentari (CUFAA), Carabinieri Force and forest services of Autonomous Regions. It represents the most harmonized and accurate of the currently available wildfire datasets for the entire Italian territory for the 2007 to 2017 time period. Along the peninsula, wildfires did not occur uniformly (Fig. 1). The most affected regions are located in Southern Italy, in particular the islands of Sardinia (17,184) and Sicily (10,450), followed by Calabria (11,087) and Campania (10,728). On the other hand, few wildfires were recorded in Northern Italy. In one decade, only 112 wildfires occurred in Valle d’Aosta, making it the region with the least number of wildfires in the entire peninsula. Furthermore, the wildfire trend was not constant throughout the peninsula. The year with the highest recorded frequency was 2007 exceeding 10,000 fires; after 2007, the frequency of wildfires significantly decreased and subsequently increased again in 2011 and 2012 registering 9209 and 9281 wildfires, respectively. The lowest frequency of the reference period was recorded in 2013 (3770 wildfires). After that time, the frequency slowly increased to reach yet another peak in 2017 with slightly fewer than 9,000 wildfires.

These data were used to create a 2-km resolution map of the ground truth (response variable). More precisely, we assigned the label “1” (fire) to each grid point if at least one wildfire ignition event occurred within that area during the considered time interval, otherwise we assigned the label “0” (no fire). As a result, we obtained a wildfire occurrence map of the Italian territory at a 2-km scale. Using a 2 km × 2 km resolution decreases the imbalance between fire and no-fire events, facilitates the learning phase and decreases the computational burden.

Following previous studies^17,18^, we collected specific features accounting for biophysical and human-related drivers from available official and non-governmental sources, such as the Corine Land Cover initiative, the Copernicus Land Monitoring Service and OSM. We employed climate and landscape data to take into account the high heterogeneity and variability of the Italian territory from north to south and from the coast to inland areas. For example, NDVI is related to vegetation characteristics, and it’s able to quantify vegetation greenness and understanding vegetation density that can potentially burn^19^. Further, we selected four human-related drivers according to the proximity to roads and railways and human presence. Many authors have proved a strong linkage between wildfire occurrence probability and human systems in Mediterranean countries of Southern Europe^20–23^. All these variables have been selected on the base of their potential relation with wildfire occurrence for the period of investigation and their availability at our scale. In particular, our dataset included twelve biophysical exploratory variables: seven land cover classes such as agriculture, forest, grass, shrubs, wetland, water, other lands, one tree cover density, the Normalized Difference Vegetation Index (NDVI), slope and elevation (DTM); four human-related variables: distance of a grid point from a human settlement, from a road and from rails, and population density and one climate Fire Weather Index (FWI)^6^. The temporal series of both FWI and NDVI were seasonally averaged, i.e., their mean values were computed for the fire seasons (from June to August) and then used as input for the subsequent analyses. Regarding the Corine Land Cover layer, we preferred to consider each class as a continuous explanatory variable (ranging from 0 to 100%) instead of building a single categorical variable. The former data representation is particularly useful when more Corine classes coexist within a resolution pixel, as in wildland urban interface areas. Further details about data preparation and homogeneity are given in Online Appendix A. Our data consist of 16 explanatory variables and 1 response variable (accounting for the presence or absence of wildfires) for a total amount of 75,298 grid points.

## Methods

### Methodological overview

**Figure 2:**
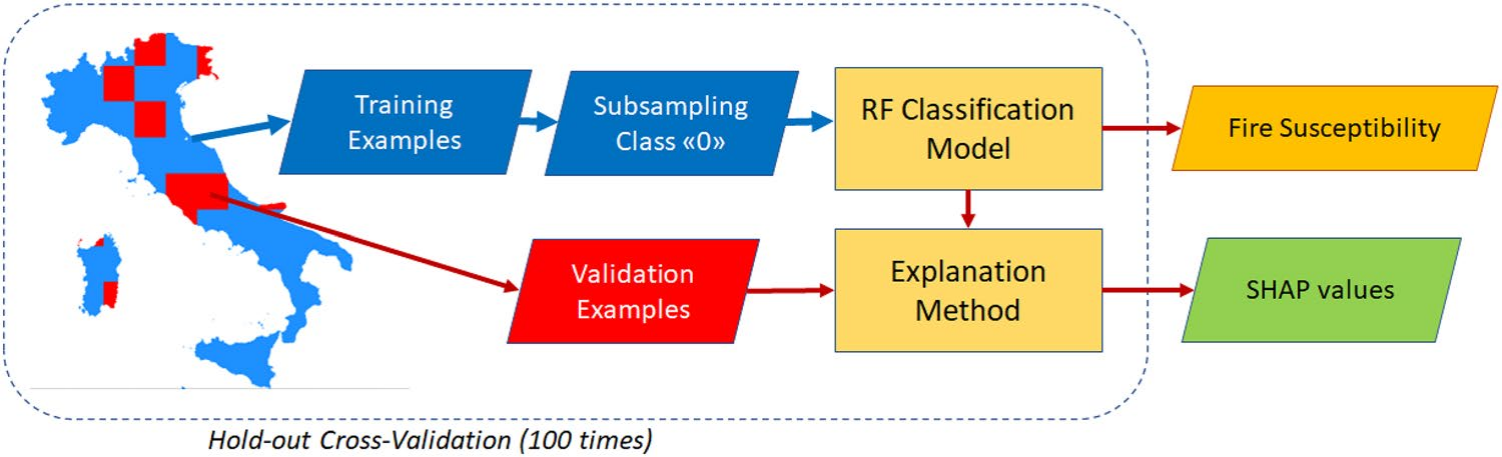
Methodological overview. Environmental, biophysical, and human-related variables are used to model wildfire occurrence over the Italian peninsula. The map was produced by using the plot.raster function from "raster" R package.

The main goal of this work is to provide an XAI framework to explain which biophysical and/or human-related factors drive wildfire events over the Italian territory (see Fig. 2 for a schematic overview). To this end, we designed a robust cross-validation framework to train a Random Forest (RF)^24^ model of Italian wildfires and evaluate the reliability of the available knowledge base; then, we performed an explainability analysis according to the Shapley paradigm^25^. Finally, we performed an independent spatial analysis using Getis-Ord statistics^26^ to investigate the presence of anomalous wildfire hotspots on the Italian territory. In the following sections, we provide further algorithmic details and insights about the processing workflow.

### Random forest detection of wildfire occurrences

**Figure 3:**
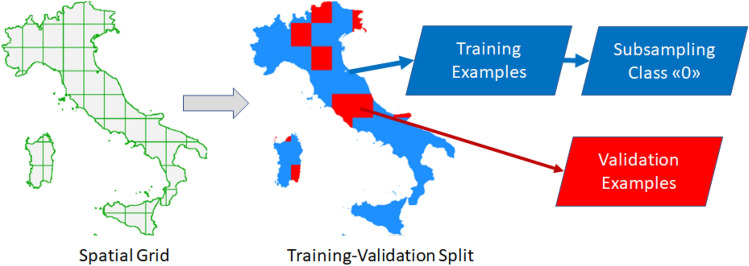
The Italian peninsula is covered by large cells (5000 km^2^ each) to perform a hold-out spatial CV procedure. Training and validation examples were assigned to different cells so that the model would not be trained on adjacent grid points (except for points along the cell borders). Furthermore, training examples were subsampled to ensure a balanced ratio between the two classes. The map was produced by using the plot.raster function from "raster" R package.

Based on the record of previous wildfires, we trained a ML classification model to predict the probability of wildfire occurrence and thus provide, through the classification score, a measurement of wildfire risk in a specific grid point. We designed a stratified 5-fold cross-validation (CV) strategy to remove spatial correlation across adjacent grid points (from here on referred to as spatial CV) and evaluated the classification performance of a RF classifier ensemble. In particular, we divided our study area into 55 cells of approximately 5000 km^2^ each (Fig. 3). Each cell includes, on average, 1500 grid points. The spatial CV was performed by considering 80% of the cells for training and 20% for validation. A random subsampling of the training examples was performed to avoid any unbalance between no-fire (“0”) and fire (“1”) grid points during the learning phase. Thus, for each CV round we made sure that, on average, the approximate 40,000 grid points used for training were not spatially close to the 15,000 grid points used for validation. To minimize spatial correlation, grid points from the same cell were not used for both training and validation, so that only border pixels (~2% of validation pixels) were subject to this condition. A cross-validation round ends when all observations have been used for validation; as the folds were randomly sampled, the procedure was repeated 100 times to ensure statistical robustness. This procedure is of paramount importance to achieve an unbiased performance estimate, a highly relevant issue in remote sensing image processing^27,28^. For instance, using two adjacent pixels for training and testing, respectively, would lead to performance overestimation as the two examples are often indistinguishable.

Wildfire probability assessment was performed by a RF model implemented with the *h2o* (v.3.34.0.3) R 3.6.3 package^29^. RF is a state-of-the-art classifier that ensures performance accuracy with a relatively simple tuning process, as the main parameters determining its performance are the number of trees in the forest and the number of features randomly selected at each node split. For this analysis, we found that with 50 trees the training error reached a stable plateau. A low number of trees is also desirable to obtain low computational processing times. With regard to the second parameter, we used the default value (i.e., the square root of the number of features); using other values did not significantly change the results.

### Evaluating model reliability

The performance of a ML model can be evaluated with several metrics appropriately selected according to the purposes of the application. However, in general, one can derive most performance metrics from the confusion matrix, a 2 × 2 matrix (for binary classification) with columns that usually indicate the predicted classes and rows that represent the actual classes. Accordingly, one can define the True Positive (TP) set consisting of correctly classified positive class elements and, analogously, the True Negative (TN), False Positive (FP) and False Negative (FN) sets. From these values a straightforward definition of performance metrics is possible, such as accuracy (*Acc*), sensitivity (*Sens*), precision (*Prec*), specificity (*Spec*) and *F*1, as shown in the following equations:

1 $$Acc = \frac{TP + TN}{{TP + TN + FP + FN}}$$

2 $$Sens = \frac{TP}{{TP + FN}}$$

3 $$Prec = \frac{TP}{{TP + FP}}$$

4 $$Spec = \frac{TN}{{TN + FP}}$$

5 $$F1 = \frac{2 \, Sens \cdot Prec}{{Sens + Prec}}$$

We considered as positive instances grid points with label “1” (fire). Accuracy is a global performance indicator while sensitivity and specificity focus on the model’s ability to detect positive and negative examples, respectively. *F*1 is another global metric recommended for unbalanced data. One drawback of previous metrics is that they all rely on a decision threshold, which is usually set at 0.5; an alternative is commonly given by the area under the Receiver Operating Characteristic (ROC) curve (AUC). Although the informative content of these metrics consistently overlaps, we provide all of them to facilitate a comparison of our findings with previous studies.

### Explaining Italian wildfires

In environmental sciences, the explainability of ML models is as important as prediction accuracy. Artificial intelligence models can be explained either at the global or local level. We estimated the global importance of a feature by measuring whether the inclusion or exclusion of the given feature from the model affected the algorithm’s performance on the validation set; in other words, we measured the importance of a feature by the difference between performance scores obtained with and without the given feature. Moreover, to deal with possible feature importance biases due to input feature distributions, we estimated the significance of importance metrics with permutation. In fact, some authors have noticed that purity-based feature importance can be biased toward continuous features with uniform distribution or high-cardinality categorical features^30^ and suggested the use of statistical tests based on permutations (PIMP method) to evaluate significant features. Here, the R package vita (v 1.0.0)^31^ was adopted.

In addition, to explain how the algorithm made a specific decision locally and, therefore, to understand which factors explain wildfire occurrence, we adopted the Shapley paradigm^32,33^. The basic idea is borrowed from game theory: the features of a model are like the players of a game; cooperation is the key to win, or in the case of a learning model, to achieve the correct decision, but it should be noted that not all players/features yield the same contribution. The SHAP value of a feature *j* is introduced to evaluate such contributions by comparing how the classification scores change by including or removing this feature (Eq. 6):

6 $$SHA{P}_{j}(\overrightarrow{x})={\sum }_{S:j\in S}{\left[\left|S\right|\times \left(\genfrac{}{}{0pt}{}{|F|}{\left|S\right|}\right)\right]}^{-1}[ {p}_{s}\left(\overrightarrow{x} \right)-{p}_{s/j}\left(\overrightarrow{x}\right)]$$

where $\overrightarrow{x}$ is an observation defined by a feature vector, the sum is intended over all the subsets *S* of features which include the feature *j*, ${p}_{s}\left(\overrightarrow{x}\right)$ denotes the classification score obtained including *j* while ${p}_{s/j}\left(\overrightarrow{x}\right)$ denotes the score obtained removing *j* from the set of features. The possible combinations for a set of features *F* with *|F|* elements and a subset *S* with |S| elements are $\left[\left|S\right|\times \left(\genfrac{}{}{0pt}{}{|F|}{\left|S\right|}\right)\right]$. Thus, given the SHAP values of all the input features (for all observations) it is possible to understand how each decision was obtained. In this study, we used the *h2o.shap_summary_plot* function implemented in the *h2o* (v.3.34.0.3) R package^29^.

### Anomalous wildfire hotspots

The results provided by the implemented ML model and its explainability were finally complemented by a spatial analysis with Getis-Ord G* statistics^26^ aimed at determining the presence of anomalous spatial patterns of wildfire events. Given an image or a map, in this case a map of wildfire events, Getis-Ord G* statistics of the i^th^ pixel are actually a *z*-score, given by the following Eq. (7):

7 $${G}_{i}^{*}=\frac{{\sum }_{j=1}^{N}{w}_{i,j}{y}_{j}-\overline{y }\left({\sum }_{j}{w}_{i,j}\right)}{S\sqrt{\frac{n {\sum }_{j=1}^{N}{w}_{i,j}^{2}-{\left( {\sum }_{j=1}^{N}{w}_{i,j}\right)}^{2}}{n-1}}}$$

where *y*_*j*_ is the total number of wildfire events that occurred during the decade investigated, *N* is the total number of instances of the dataset, *w*_*i,j*_ is a spatial weight accounting for spatial proximity of the pixels i and j (Eq. 8):

8 $$\begin{gathered} \overline{y} = \mathop \sum \limits_{j = 1}^{N} x_{j} /N \hfill \\ S = \sqrt {\mathop \sum \limits_{j = 1}^{N} y_{j}^{2} /N - \overline{y}^{2} } \hfill \\ \end{gathered}$$

For the present study we chose *w*_*i,j*_ as the inverse of the Euclidean distance between a pair of pixels i and j. Getis-Ord G* statistics are useful when investigating spatial processes, since they can assess local spatial independence of stochastic events or the emergence of hotspots. Significant hotspots of a spatial process can be identified as those pixels that have high values of G* statistics, namely G* > 2^26^; in this study, we used Getis-Ord G* spatial statistics from the *spdep* R package^27^.

## Results

### Reliability of the wildfire occurrence model

To evaluate the informative content provided by the available variables and the extent to which they are able to accurately describe wildfire susceptibility, we performed a binary classification and compared the CV performance with the out-of-bag error (without the spatial-bias correction) in Table 1. The difference between the two columns accounts for the spatial correlation bias and also returns an evaluation of the maximum performance achievable.

**Table 1:** The medians and 95% confidence intervals for all adopted metrics are reported and out-of-bag (OOB) performances of models with and without spatial cross-validation (CV) are compared.

Metric (%)	OOB Performance	Spatial CV
*Acc*	72.9 (72.1, 73.3)	69.7 (63.6, 75.7)
*F*1	65.1 (64.3, 65.7)	62.0 (53.2, 69.0)
*Sens*	82.5 (81.8, 83.6)	78.7 (69.9, 87.8)
*Prec*	53.7 (52.8, 54.5)	50.9 (43.0, 59.1)
*Spec*	68.7 (67.6, 69.2)	69.2 (48.9, 76.0)
*AUC*	84.1 (83.8, 84.5)	81.3 (76.0, 84.8)

We evaluated our machine learning models in terms of accuracy (*Acc*), *F*1 score, sensitivity (*Sens*), precision (*Prec*), specificity (*Spec*) and area under the ROC curve (*AUC*).

Classification performances in terms of AUC and accuracy were 81*.*3 and 69*.*7%, respectively. We observed a significant drop in *F*1 (62*.*0%), which attests to the difficulty of detecting wildfires. This is reflected by a precision of 50*.*9%, which implies that in half of the cases a predicted wildfire did not match an actual wildfire; however, the resulting high sensitivity denotes the effectiveness of the method in detecting wildfires. By comparing the performance of the model without the spatial CV strategy with the performance of the model with spatial CV strategy, we found an almost negligible performance increment (only 3.2% in terms of accuracy).

### Outlining the features associated to wildfire occurrence

**Figure 4:**
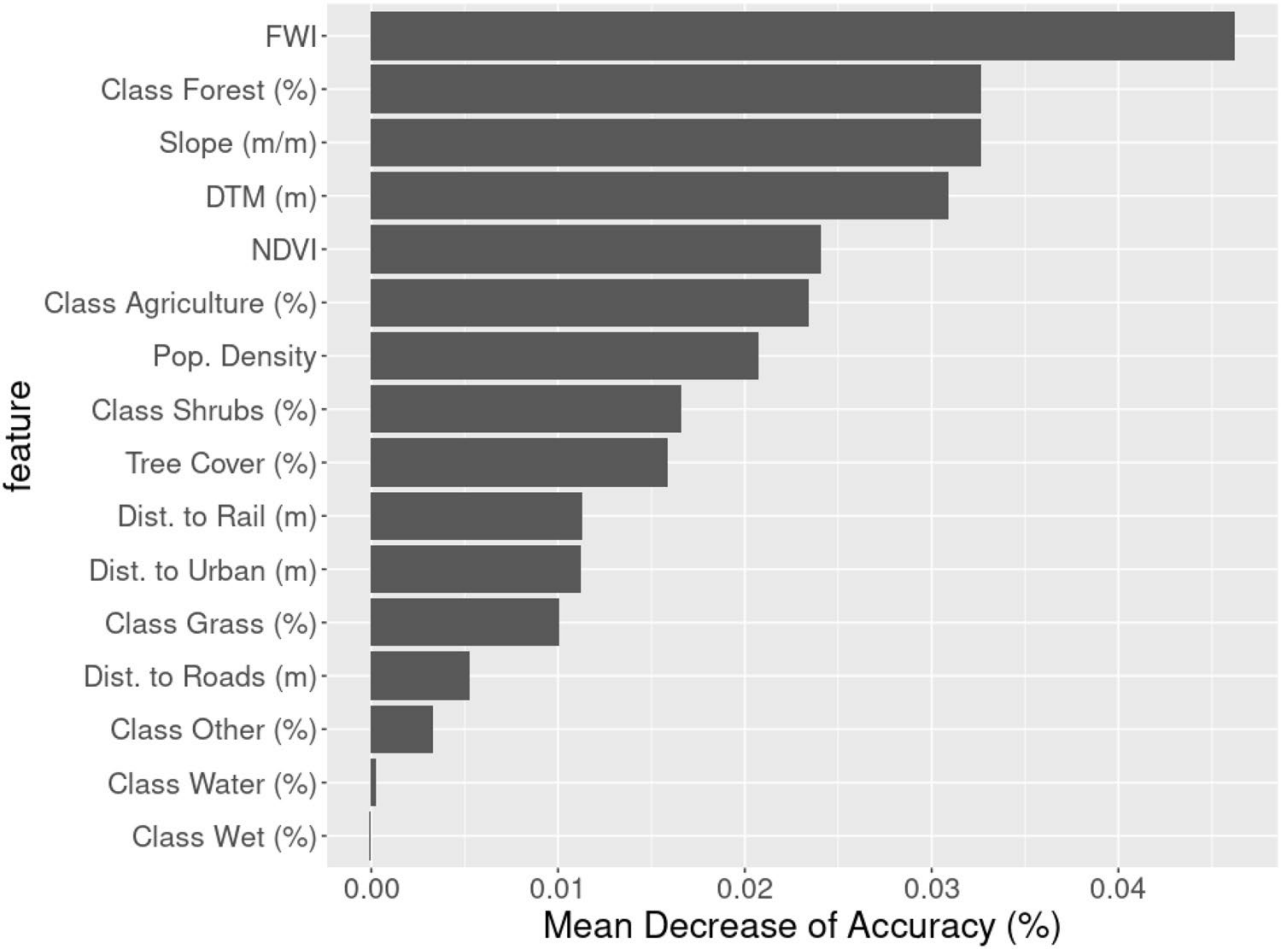
Feature importance embedded in the Random Forest model in terms of mean decrease in accuracy.

Once model reliability was demonstrated, we investigated the global importance of the available features. The goal of this analysis was to identify the most important factors for wildfire occurrence prediction; the results are presented in Fig. 4. The most important features were FWI, “Forest” class, Slope and DTM. To establish the number of features significantly associated with wildfire risk prediction, we used the PIMP method. All variables except the “Wet” class were significant (p-value < 0*.*01).

### Explaining wildfire drivers

**Figure 5:**
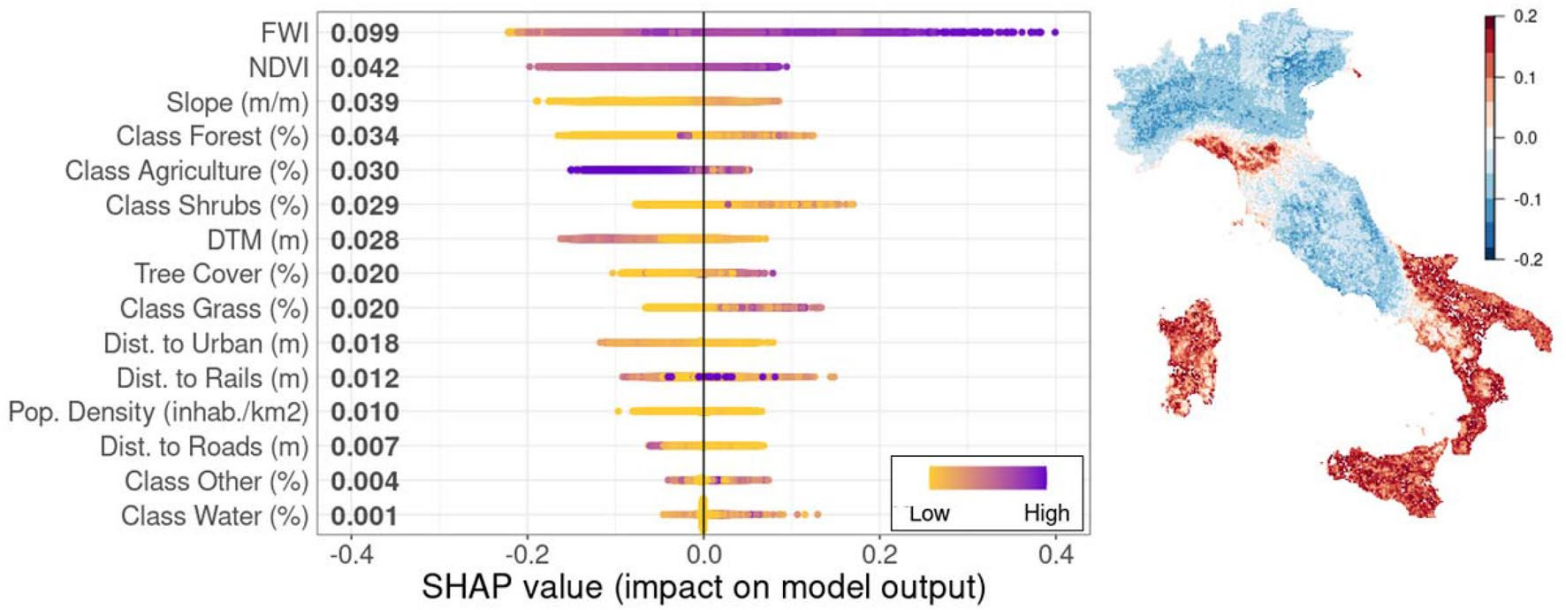
(Left panel) Shapley values for wildfire occurrence prediction. On the y-axis, the features are sorted by decreasing importance while the x-axis shows the SHAP value distribution and denotes whether a variable contributes to reduce or to increase the fire probability. Gradient colors indicate the original value for that variable. Each point represents a row from the original dataset. (Right panel) Fire Weather Index (FWI) Shapley values. For sake of readability, SHAP values exceeding the [− 0.2, 0.2] range were assigned the same colors of extreme points of the chosen interval. The map was produced by using the plot.raster function from "raster" R package.

Feature importance is key to understanding which factors can support wildfire occurrence prediction. Further insight is provided by explainability analysis that evaluates how features contribute to the classification score, in our case wildfire occurrence. Results of this analysis are presented in the left panel of Fig. 5. Although all variables were significant except the “Wet” class, Shapley values provided a sharper insight, i.e., that wildfire occurrence is determined by one main feature—the FWI. Accordingly, we show the map of Shapley values in the right panel of Fig. 5.

**Figure 6:**
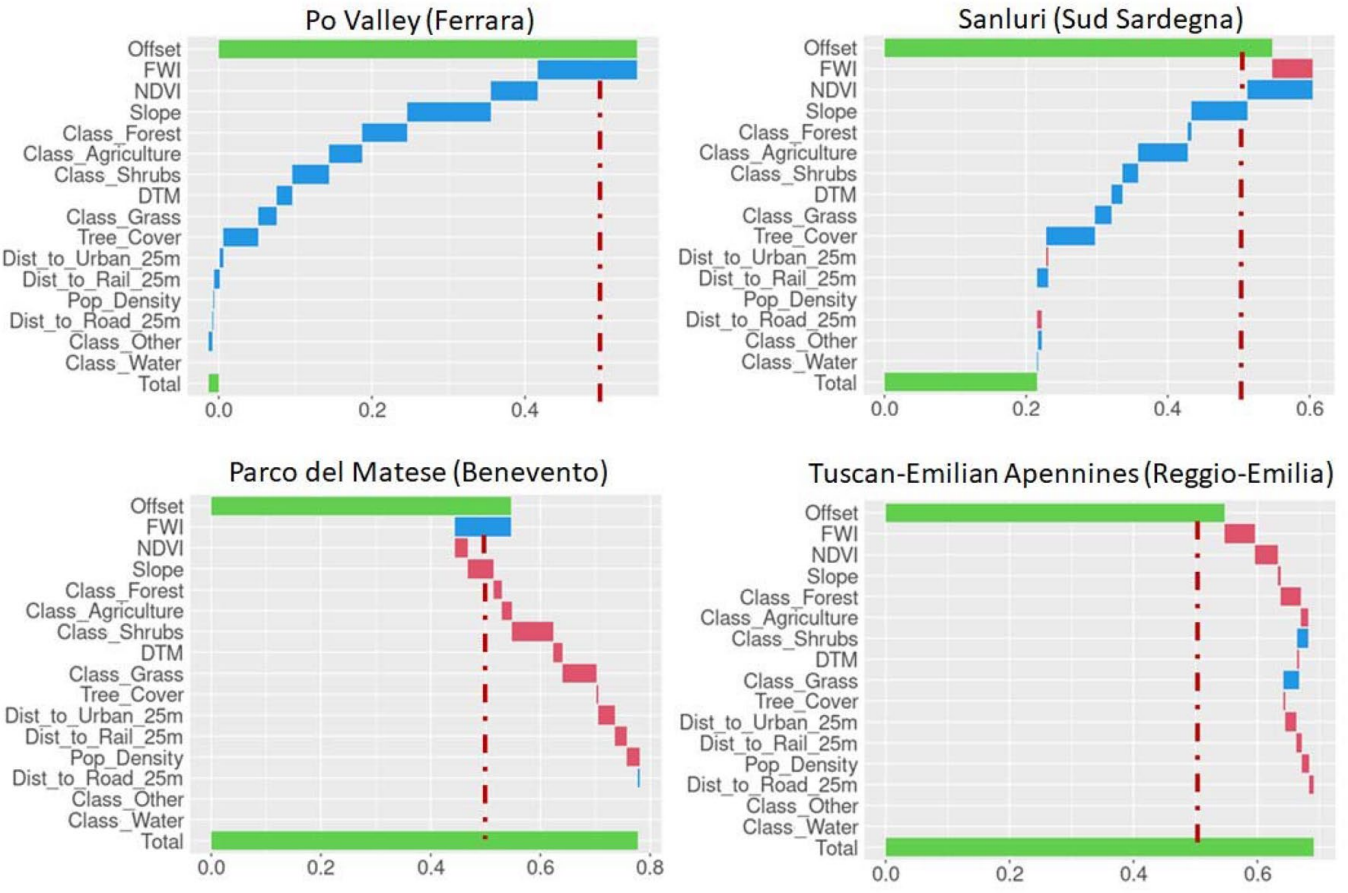
Explainability and classification results of 4 notable cases: Po Valley—True Negative (top left panel); Campidano Plain near Sanluri, South Sardinia—False Negative (top right panel); Parco del Matese, province of Benevento—True Positive (bottom left panel); Tuscan-Emilian Apennines—False Positive (bottom right panel). Fire Weather Index (FWI), Normalized Difference Vegetation Index (NDVI), Digital Terrain Model (DTM).

Global analyses yield general information about how features affect the model; further information can be obtained by observing how SHAP values are related to feature values. In particular, Fig. 5 shows that both high and low feature values contribute to positive and negative SHAP values, accordingly. Partial dependence plots showing how these values are related are provided in Online Appendix B. Moreover, it is important to evaluate what happens case by case, as the geographical specificities, political decisions or other factors make each location different from the other. For example, the fact that the FWI is globally the most important feature does not ensure that in a specific situation the greatest contribution to fire susceptibility can be given by NDVI, or slope, or another factor. Locally, explainability analyses allow to directly identify how wildfire occurrence is computed in terms of the available features. Figure 6 shows the waterfall plots for four notable cases: the Po Valley (near Ferrara), the Campidano Plain (South Sardinia), Parco del Matese (province of Benevento), and the Tuscan-Emilian Apennines (Reggio-Emilia). These cases are meant to illustrate the behavior of this type of representation for True Positive, False Positive, False Negative and True Negative classifications.

Waterfall plots should be read from top to bottom. The top green bar represents the Shapley base value (Offset), i.e., the mean probability value on the training examples (0.53). The bottom green bar corresponds to the probability score (Total) for that specific grid point and is the sum of the Shapley values and the Offset. The horizontal bars between Offset and Total correspond to the Shapley values, namely the contribution of each feature to the overall classification score. The features depicted in red and blue bars increase and decrease the overall classification score, respectively. The vertical dashed line corresponds to the classification threshold (0.5). If the Total classification score is greater than the threshold, the observation will be classified as “1” (fire), otherwise it will be classified as “0” (no fire).

Considering the waterfall plot for the Po Valley near Ferrara (top left of Fig. 6), all features contributed to a decrease in the classification score from Offset; as a result the overall classification score (Total) is almost zero. The greatest (negative) contribution in magnitude is given by FWI and Slope.

The Tuscan-Emilian Apennines (bottom right of Fig. 6) is an example of location where the FWI feature positively contributed to the overall classification score (around 0.7), together with NDVI and Forest. The RF model assigned a “1” label (fire) to this case. In the other selected cases in Fig. 5, the algorithm returned incorrect predictions, and the waterfall plot describes how each variable contributed to the final classification score.

### Detection of wildfire clusters with Getis-Ord G* statistics

Finally, we detected wildfire hotspots according to Getis-Ord G* statistics. Most of the hotspots are concentrated in Southern Italy, with a few anomalous wildfires in the North. These are mainly found in the Liguria region and in the Friuli–Venezia Giulia region on the border with Slovenia, while the greatest concentration of hotspots in Southern Italy occurs in Sardinia and along the Tyrrhenian coast of the Campania and Calabria regions. The presence of hotspots in Apulia and Sardinia is discreet. Five regions of national territory do not present hotspots (i.e., Aosta Valley, Trentino—South Tyrol, Emilia–Romagna, Marche, and Umbria), whereas the region with the highest number of hotspots is Sardinia, as shown in Fig. A2.

**Figure 7:**
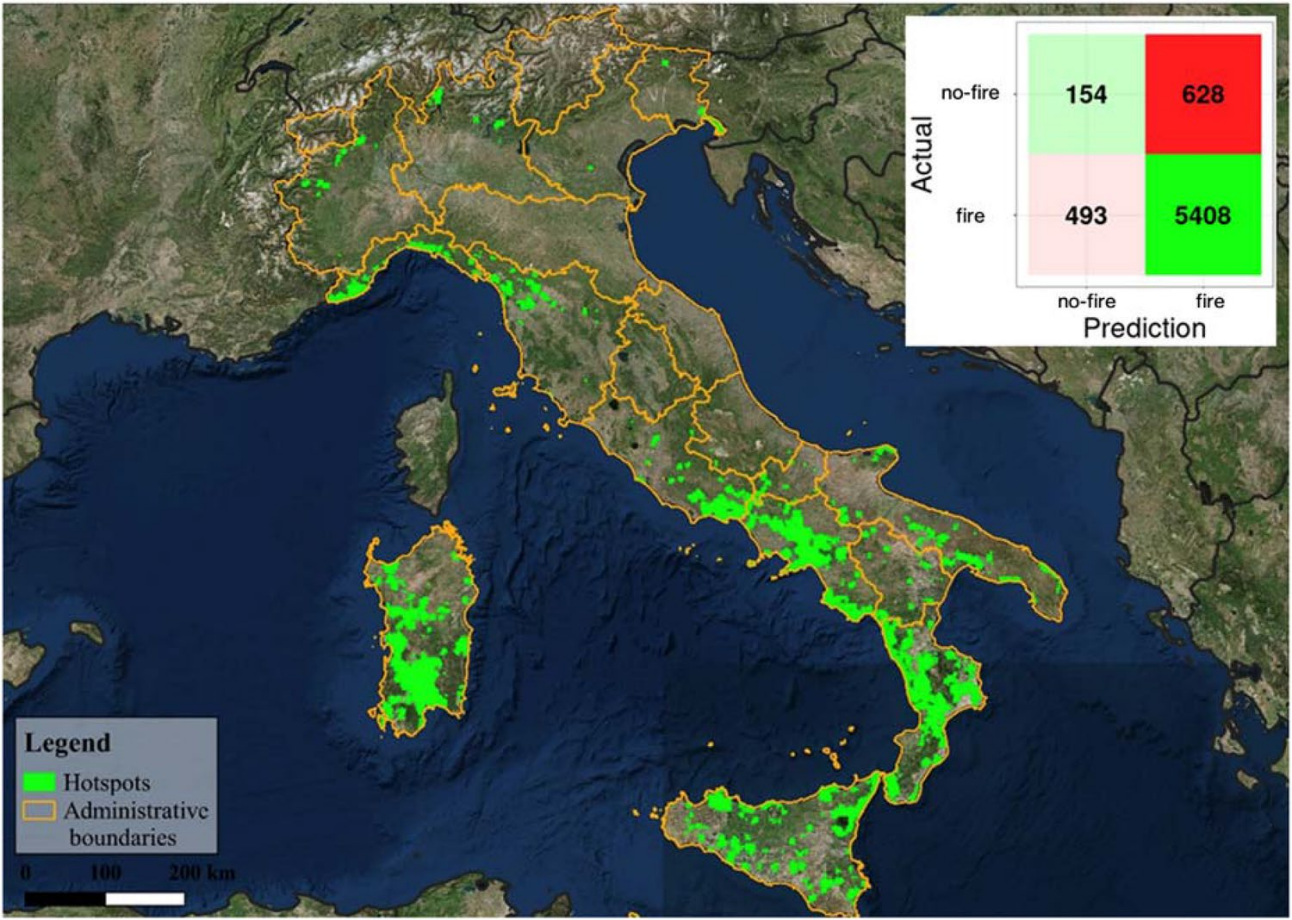
Spatial distribution of wildfire hotspots according to Getis-Ord G* statistics at a 95% confidence interval. Confusion matrix between RF predictions and actual binary labels (top right grid). The green scale for diagonal terms and red scale for off-diagonal terms in the confusion matrix emphasize the impact of the matrix terms on the overall ratio. The map was produced on QGIS using the "new layout" feature. The map was produced by using the plot.raster function from "raster" R package.

Our analysis detected 6683 hotspot pixels from a total of 75,298 observations at a 95% confidence interval (Fig. 7). Of these hotspots, 782 (11%) belong to class “0” (no-fire) while 5901 (89%) belong to class “1” (fire). Worth noting is the possibility to obtain G positive values with no-fire pixels observed because of the internal smoothing of this statistic, which involves adjacent points weighted according to their distance. Our RF model identified 6032 positives, of which 5408 are true positives (Fig. 7, right panel). More than half of the wildfire hotspots that were wrongly classified as class “0” events are located in the Campidano plain in Sardinia. The remaining spatial clusters of False Negative hotspots are limited in extension and spread throughout the Italian peninsula.

## Discussion

The main intent of our study was to present a comprehensive framework to determine wildfire occurrence and its drivers. As a case study of particular interest, we examined the Italian peninsula given its heterogeneity in terms of ecological, climatic and environmental features. To this aim, we evaluated the extent to which wildfire occurrence can be estimated based on environmental, biophysical and human-related indicators accounting for territorial morphology or the presence of settlements and models of transport, among others. Initially, we estimated classification performances and obtained values which are comparable with those of previous studies^15,16^ (e.g., AUC 81.3%). On the one hand, this result confirms that wildfire occurrence can be robustly predicted, as analogous performances have been achieved by independent studies with different designs, algorithms, and data. On the other hand, wildfire prediction remains extremely challenging; in fact, the maximum AUC value obtained when a simple CV strategy is adopted (84.1%), and not a spatial CV, could suggest that there is room for improvement, for example, by including additional explanatory variables. However, it should be kept in mind that the high stochasticity of wildfires, given by factors “external” to the model such as arsons, will continue to make certain events totally unpredictable. Nonetheless, as the classification performances demonstrate, the available indicators provide a reliable knowledge base for wildfire risk modeling.

XAI fully explores the factors driving wildfire probability at regional and local scales. According to our knowledge, this study represents the first that uses XAI techniques applied to wildfires. The use of XAI at regional and local scales highlights the driving factors of wildfires and, in principle, can be an important asset to assist the management and monitoring of environmental areas at risk. Moreover, the possibility to identify wildfire driving factors at local scale can also support the adoption of environmental prevention and conservation policies. In particular, for the present case, the primary role played by the Fire Weather Index was revealed. Other important factors are “Slope” and “Forest”; however, it is worth noting that all the considered explanatory variables were significant according to the PIMP analyses. While it is not surprising that PIMP and SHAP analyses return different rankings (e.g., the former method is evaluated on training, the second one on validation examples), the two feature importance rankings are in good agreement: both methods reveal the primary role played by the Fire Weather Index and the negligible impact of ‘’Class_Wet’’.

As shown by several studies, the conditions related to climate change such as severe heat and drought favor wildfires^34–38^. If we do not disrupt the warming cycle, more and severe wildfires can be expected in the years ahead. Our results are in line with this general consensus. For example, Elia et al. (2022)^37^ detected a climate gradient explaining the variability of wildfire dynamics across the Italian peninsula from North to South. High exposure to warm temperatures and dry winds render wildland fuels prone to ignition, especially in the Mediterranean landscapes of Southern Europe^39–41^. Furthermore, our findings confirm the key role of land cover and human pressure on wildfire occurrence. The man-made system has been assessed by various authors as an important pool of different sources of fire ignition^42–45^. The unsound management of tourist activities, silvopastoral practices and renewal of pastures, or simply the abandonment of old fields create the appropriate conditions for wildfire ignition and spread. Our study is consistent with that of Mancini et al.^44^, who found that the frequency of wildfire events increases proportionally with the proximity to built-up areas in Italy, similarly to the findings of other studies^46–48^ for other countries of Southern Europe.

Coupling human and natural systems is the foundation for developing studies and research in the field of wildfires and, more broadly, environmental disasters. In particular, Italy as well as other Mediterranean countries of Southern Europe are characterized by a considerable heterogeneity of landscapes and ecosystems due to their complex geological histories, climate regimes and anthropic influence. These factors have led us to conduct an in-depth investigation to detect outstanding wildfire hotspots using Getis-Ord statistics. The distribution of hotspots follows different spatial patterns across the Italian territory from North to South. The North-West coastal area is characterized by several hotspots, while the Eastern zone of the Alpine area presents a reduced number of clusters. Many authors found that the alpine and subalpine Italian regions are not particularly prone to wildfire events compared to the rest of the Mediterranean landscape^49^. The use of fires in the alpine context is rare and characterized by a high level of control by the local population. Moreover, less fire-prone vegetation and weather generally slow down wildfire ignition and spread in most parts of the Italian Alps^49–51^. However, we have spotted some protected areas in the subalpine forests where wildfires occur mainly in winter, probably due to extensive tourist pressure^52^. In southern regions, we detected hotspots along the southwest coastal landscapes where the occurrence of wildfires could be potentially imputed to arson activity. On the islands of Sardinia and Sicily, traditional agricultural practices/management (burning of stubble and pruning residues) are the main cause of hotspots, more than in other territories; therefore agricultural lands tend to be increasingly affected by fires^51,53^. This result is consistent with those of Bajocco et al.^51^ and D’Este et al.^53^ denoting an opposite trend compared to the rest of Italy. The authors found that in this context, wildfires seem to have a preference for managed areas (e.g., agriculture lands) instead of wildland ecosystems. Hence, findings related to Sicily and Sardinia do not support the theory of climate change as the unique important wildfire driver. The issue may need to be assessed from a socioeconomic point of view, supporting the hypotheses related, for example, to demographic trends, education, and gross domestic product, which is not within the scope of our study.

## Conclusions

In this study we present an XAI framework for wildfire occurrence assessment in a Mediterranean landscape of Southern Europe. The results demonstrate that in such a heterogeneous territory as the Italian peninsula, the framework is efficient and statistically robust for analyzing wildfire occurrence. Consistency was found with other studies in identifying climate as the main driver of wildfires, even if the model was effective in discriminating areas where other drivers play a key role in shaping wildfire occurrence. However, our results are of secondary importance to the approach we have devised. The study aims to enrich the scientific literature on artificial intelligence applied to such stochastic natural disasters as wildfires. This field of research still has much room for improvement. Our study intended to corroborate the ever-growing body of scientific evidence on artificial intelligence and to encourage new research and projects on the operational use of machine learning models, as they are yet viewed with skepticism by insiders and decision makers. In contexts where the human and natural spheres constantly intermingle and interact, the XAI framework, suitably integrated with decision support systems, could assist forest managers to prevent and mitigate future disasters and develop strategies for effective fire management, response, recovery, and resilience.

## Supplementary Information

Supplementary Information 1.

Supplementary Information 2.

## Data Availability

The data used in this paper are publicly available from online repositories. Further details are available from the corresponding author upon reasonable request. All the maps provided in the paper and in the supplementary materials were created using open-source software QGIS 3.22.4 and R 4.1.2 (“raster” package v3.5.21). URL links to open-source software: QGIS 3.22.4—https://blog.qgis.org/2021/10/30/qgis-3-22-bialowieza-is-released/; R 4.1.2—https://cran.r-project.org/bin/linux/ubuntu/fullREADME.html; “raster” package v3.5.21—https://cran.r-project.org/web/packages/raster/index.html.
