# Supplementary material for: Explainable artificial intelligence (XAI) detects wildfire occurrence in the Mediterranean countries of Southern Europe
Source: Sci Rep. 2022 Sep 29;12:16349. doi: 10.1038/s41598-022-20347-9 (PMC9523070; doi:10.1038/s41598-022-20347-9)
Supplement: Supplementary file 1 — Supplementary Information 1. [file 41598_2022_20347_MOESM1_ESM.docx]

**Appendix A**

**List of Abbreviations**

| **Acc** | Accuracy |
| --- | --- |
| **AUC** | Area under the Receiver-Operating Characteristic (ROC) Curve |
| **CUFAA** | Comando Unità Forestali, Ambientali e Agroalimentari (Italian Forest Services) |
| **CV** | Cross-validation |
| **DTM** | Digital Terrain Model |
| **ESA** | European Space Agency |
| **F1** | F1-score |
| **FN** | False Negative |
| **FP** | False Positive |
| **FWI** | Fire Weather Index |
| **ML** | Machine Learning |
| **NDVI** | Normalized Difference Vegetation Index |
| **OOB** | Out-of-Bag error |
| **OSM** | Open Street Map |
| **PIMP** | Permutation Feature Importance with p-values |
| **Prec** | Precision |
| **RF** | Random Forest |
| **ROC** | Receiver-Operating Characteristic Curve |
| **Sens** | Sensitivity |
| **Spec** | Specificity |
| **TN** | True Negative |
| **TP** | True Positive |
| **XAI** | eXplainable Artificial Intelligence |

**Table A1. Summary and coding of biophysical, human-related and climatic variables employed in the study**

| Data | Input | Source | Output | Coding | Resolution/scale |
| --- | --- | --- | --- | --- | --- |
| Climate | Relative humidity (%) |  |  |  |  |
|  | Absolute maximum temperature (°C) | SCIA - ISPRA Elia et al. (2020) | Fire weather Index | FWI | 1km |
|  | Maximum wind (m/s) |  |  |  |  |
| Biophysical | Corine Land Cover | Copernicus Program | Corine Classes Percentage | Class Agriculture, Class Shrubs,Class Forest,Class Wet, Class Water,Class Grass, Class Other | 1:50000 |
|  | Tree cover density (%) | Copernicus Program | Tree canopy Percentage | Tree Cover | 100 m |
|  | Vegetation Index | MODIS | Normalized Difference Vegetetion Index | NDVI | 500 m |
|  | DTM | National Geoportal | Digital elevation map | DTM, slope | 25m |
|  | Slope (%) |  | Slope map |  |  |
| Human-related | Road maps |  |  |  |  |
|  | Rail maps | Open Street maps | Distance from roads, settlements and rails | Dist. to Roads, Dist. to Urban, Dist. to Rails | 1:50000 |
|  | Settlement locations |  |  |  |  |
|  | Population | Gallego et al. (2010) | Population density map | Pop.Density | 100 m |

**
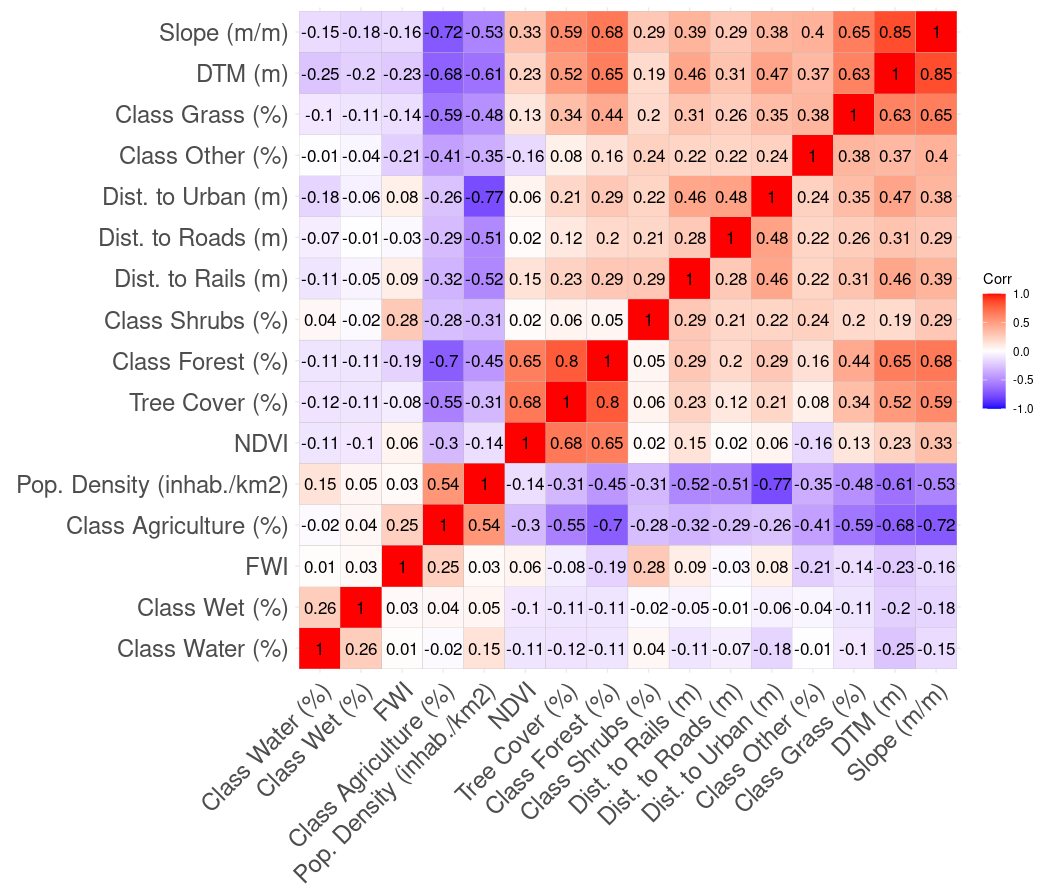
**

**Figure A.1**. Spearman's Correlation Matrix of the explanatory variables adopted for the present study. Features are ordered according to hierarchical clustering of the correlation matrix (Ward algorithm).

**
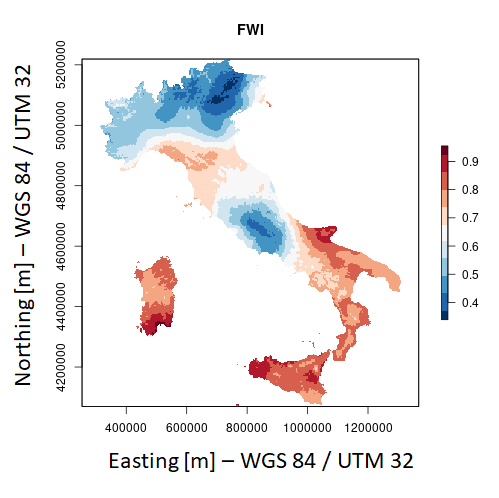
**

**Figure A.2**. Map of the summer average Fire Weather Index (FWI) computed for the period of interest (years from 2007 to 2017). High FWI values are related to fire-prone weather during summer. Unit measure: dimensionless.

**
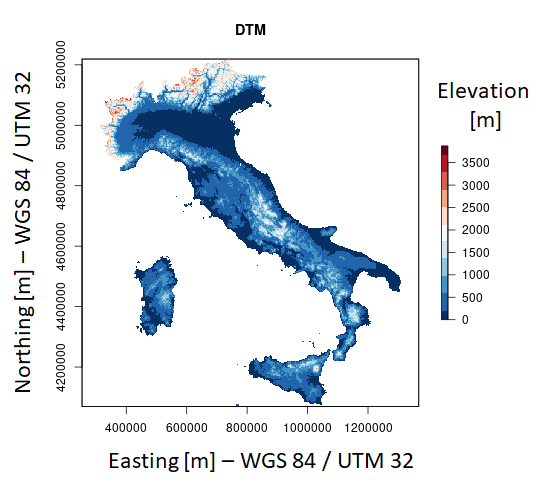
**

**Figure A.3**. Map of Elevations (Digital Topographic Map, DTM) in Italy. Unit measure: meters.

**
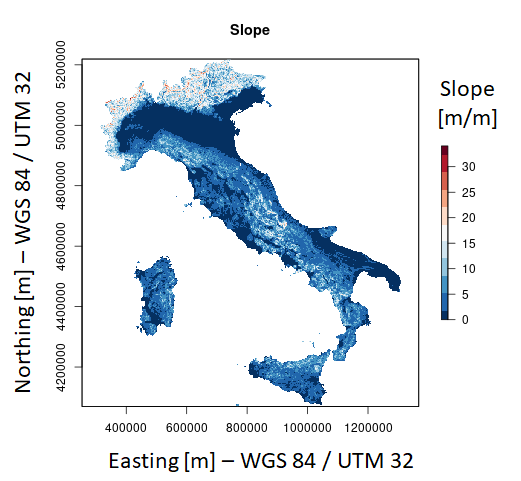
**

**Figure A.4**. Map of the Elevation Slope. Unit measures: %.

**
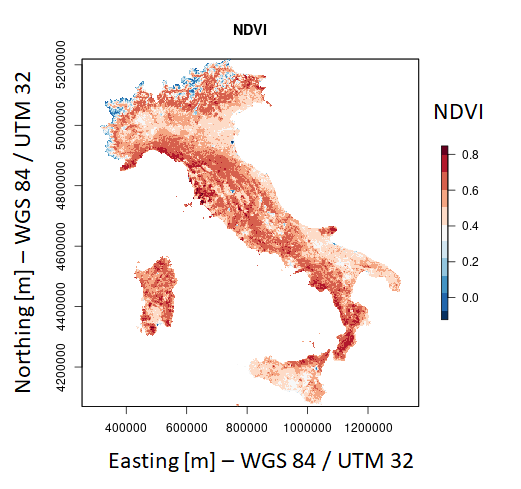
**

**Figure A.5**. Map of summer average Normalized Difference Vegetation Index (NDVI) computed for the period of study (years from 2007 to 2017). The NDVI ranges from -1 to 1. High values of NDVI (0.7-1.0) are related to dense and healthy vegetated area; from 0.4 to 0.7 are related to sparsely vegetated areas or crops; and from -0.2 to 0.3 are related to bare soils and rocks, snow, and ice. Unite measures: dimensionless.

**
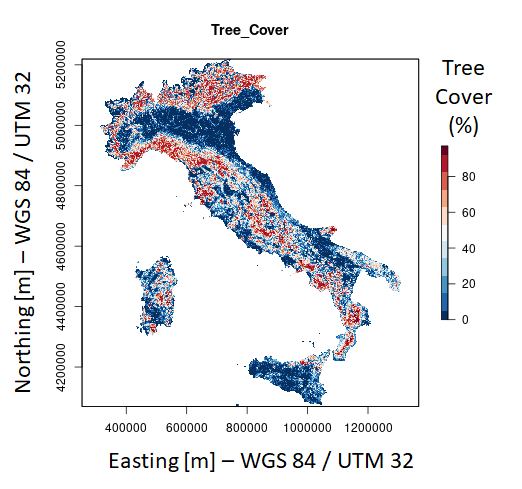
**

**Figure A.6**. Map of Tree Cover Area (%), defined as the percentage of tree cover per unit area. Tree cover ranges from 0% for bare soils, urbanized areas, and crops, to 100% for forests.

**
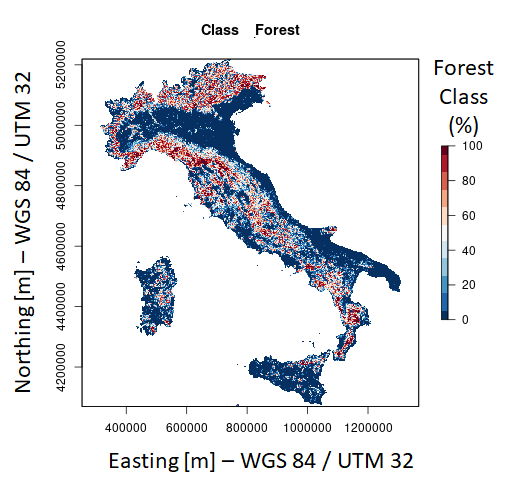
**

**Figure A.7**. Map of the Corine 2012 Land Cover Forest Class percentage.

**
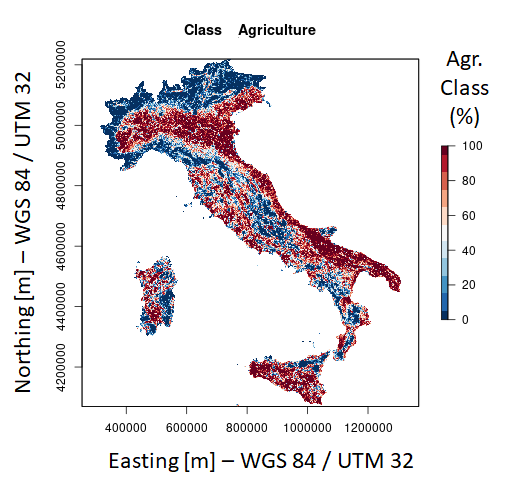
**

**Figure A.8.** Map of the Corine 2012 Land Cover Agriculture Class percentage.

**
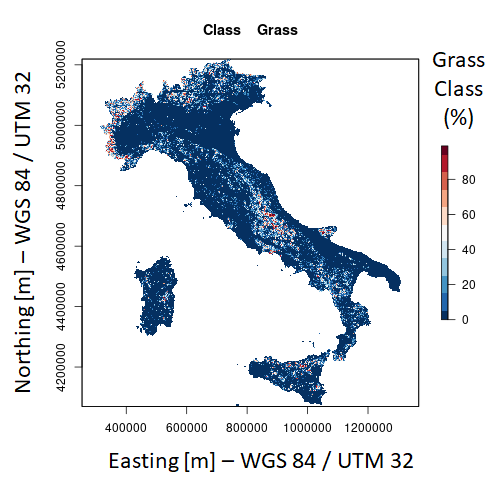
**

**Figure A.9.** Map of the Corine 2012 Land Cover Grass Class percentage.

**
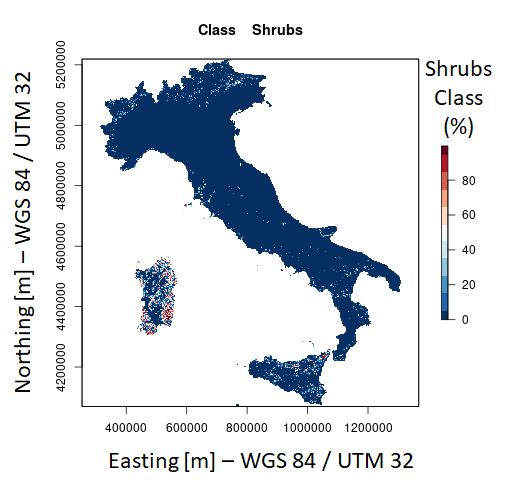
**

**Figure A.10.** Map of the Corine 2012 Land Cover Shrubs Class percentage.

**
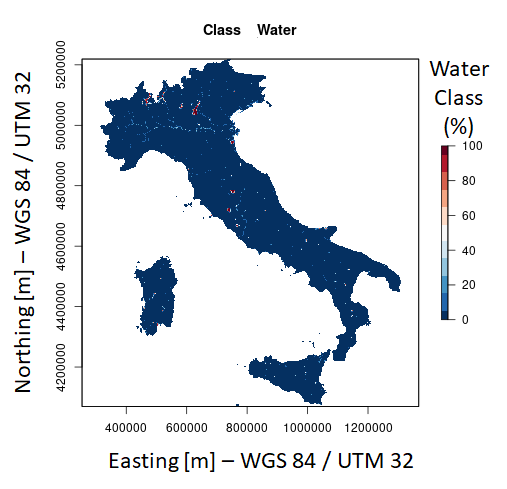
**

**Figure A.11.** Map of the Corine 2012 Land Cover Water Class percentage.

**
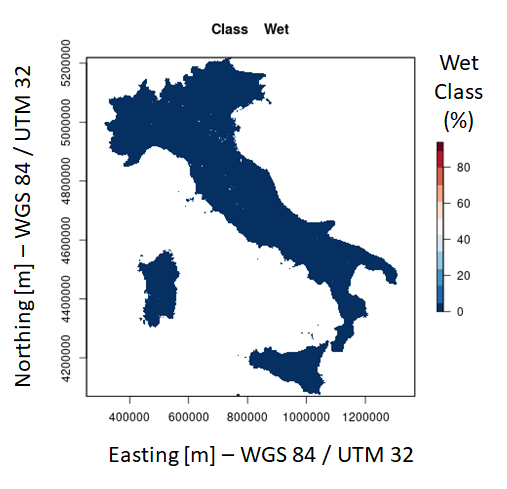
**

**Figure A.12.** Map of the Corine 2012 Land Cover Wet Class percentage.

**
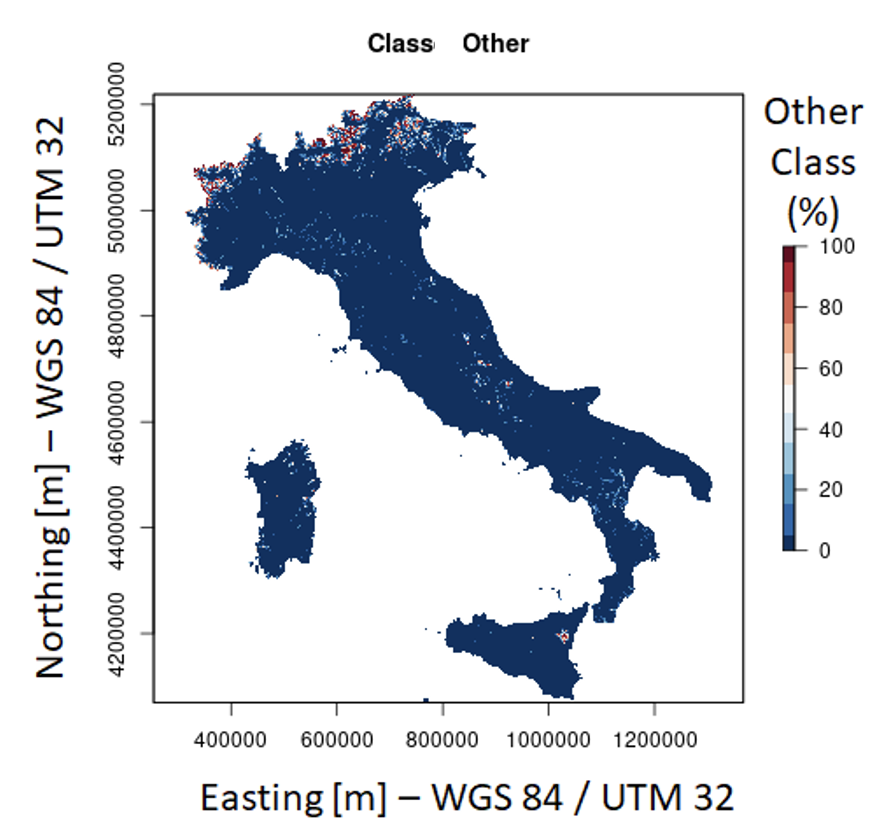
**

**Figure A.13.** Map of the Corine 2012 Land Cover “Other” Class percentage.

**
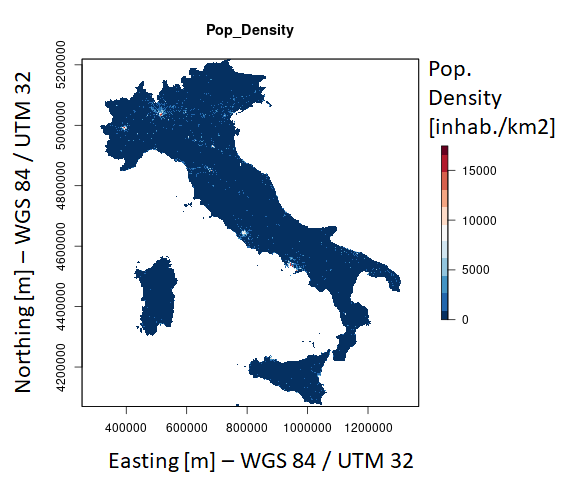
**

**Figure A.14.** Map of the Population Density in the Italian Peninsula. Unit Measure: inhabitants per square kilometers (inhabit./km^2^).

**
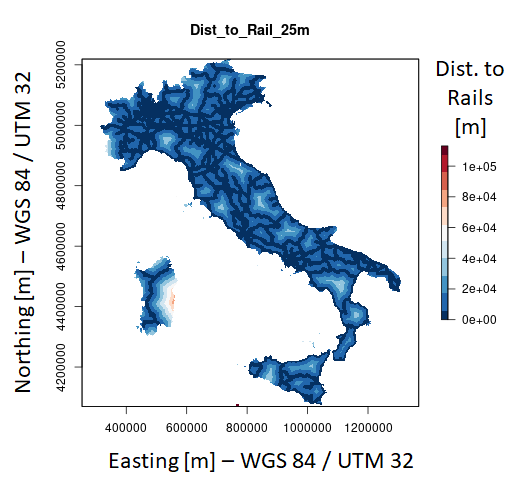
**

**Figure A.15.** Map of the Euclidean distance from the railway systems in meters. Source: Open Street Maps.


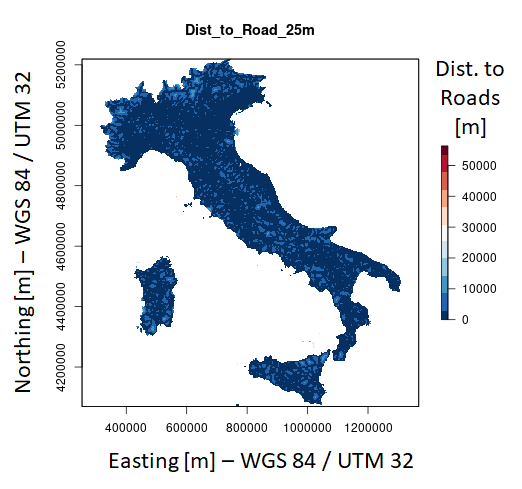


**Figure A.16.** Map of the Euclidean distance from roads in meters. Source: Open Street Maps.

**
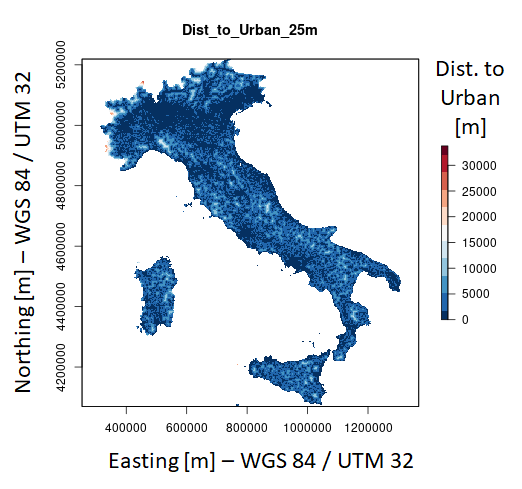
**

**Figure A.17.** Map of the Euclidean distance from urban settlements in meters. Source: Open Street Maps.

**Additional Information**

All the maps provided in the paper and in the supplementary materials were created using open-source software QGIS 3.22.4 and R 4.1.2 (“raster” package v3.5.21). URL links to open-source software:

QGIS 3.22.4 -- https://blog.qgis.org/2021/10/30/qgis-3-22-bialowieza-is-released/

R 4.1.2 -- https://cran.r-project.org/bin/linux/ubuntu/fullREADME.html

“raster” package v3.5.21 -- https://cran.r-project.org/web/packages/raster/index.html
