# Supplementary material for: Explainable artificial intelligence (XAI) detects wildfire occurrence in the Mediterranean countries of Southern Europe
Source: Sci Rep. 2022 Sep 29;12:16349. doi: 10.1038/s41598-022-20347-9 (PMC9523070; doi:10.1038/s41598-022-20347-9)
Supplement: Supplementary file 2 — Supplementary Information 2. [file 41598_2022_20347_MOESM2_ESM.docx]

**Appendix B**

**
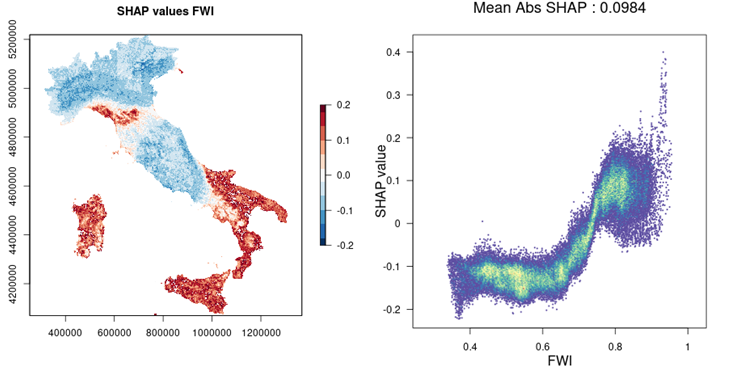
**

**Figure B.1.** [Left panel] Map of the Fire Weather Index (FWI) Shapley values. SHAP values exceeding the [-0.2,0.2] range were assigned the same colors of extreme points of the chosen interval. [Right panel] Partial correlation plot computed by plotting the FWI on the x-axis against the FWI Shapley values on the y-axis.

**
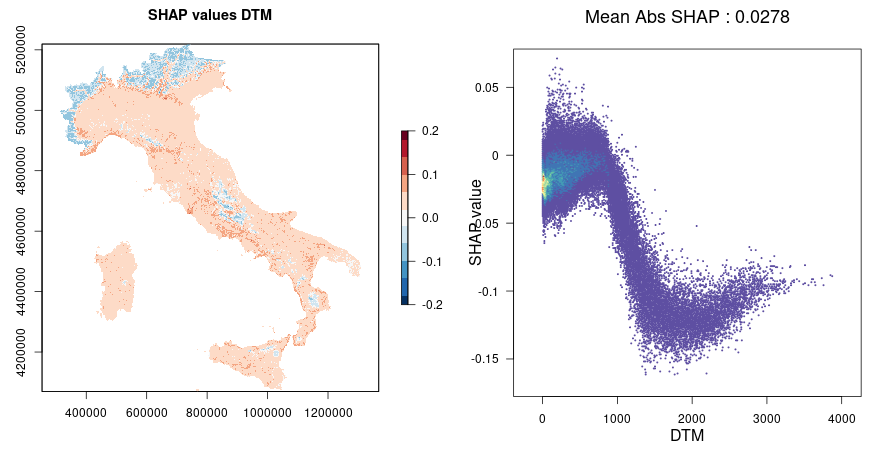
b**

**Figure B.2.** [Left panel] Map of the Digital Terrain Model (DTM) Shapley values. SHAP values exceeding the [-0.2,0.2] range were assigned the same colors of extreme points of the chosen interval. [Right panel] Partial correlation plot computed by plotting the DTM on the x-axis against the DTM Shapley values on the y-axis.

**
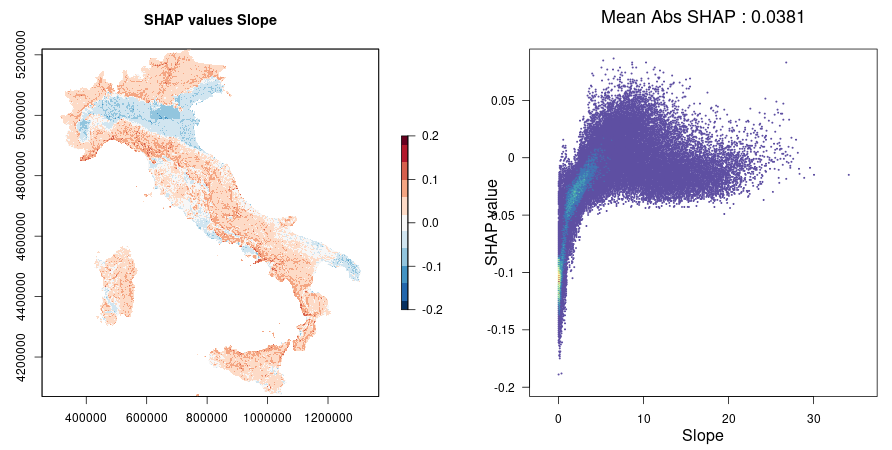
**

**Figure B.3.** [Left panel] Map of the Slope Shapley values. SHAP values exceeding the [-0.2,0.2] range were assigned the same colors of extreme points of the chosen interval. [Right panel] Partial correlation plot computed by plotting the Slope on the x-axis against the Slope Shapley values on the y-axis.

**
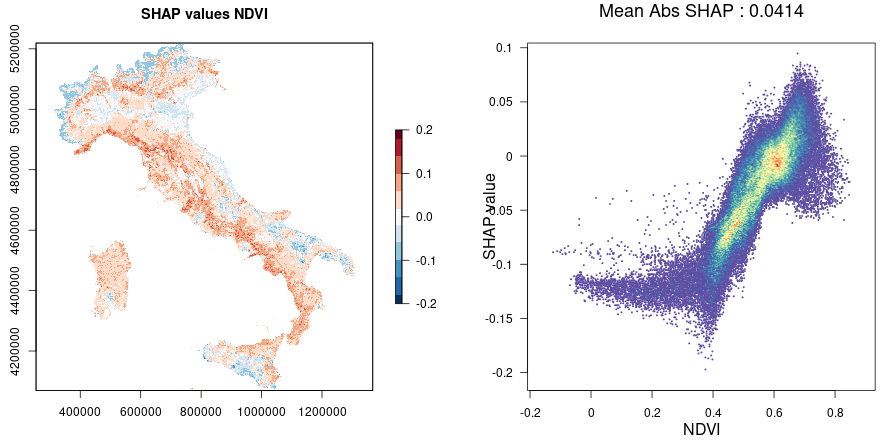
**

**Figure B.4.** [Left panel] Map of the Normalized Difference Vegetation Index (NDVI) Shapley values. SHAP values exceeding the [-0.2,0.2] range were assigned the same colors of extreme points of the chosen interval. [Right panel] Partial correlation plot computed by plotting the NDVI on the x-axis against the NDVI Shapley values on the y-axis.

**
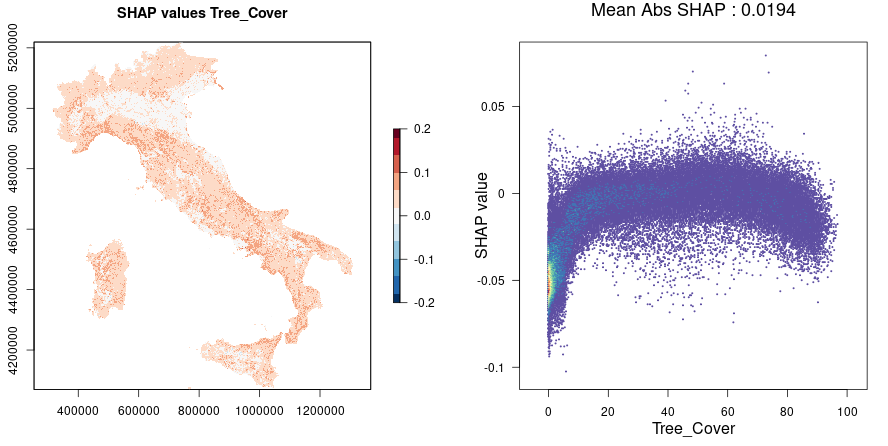
**

**Figure B.5.** [Left panel] Map of the Tree Cover Shapley values. SHAP values exceeding the [-0.2,0.2] range were assigned the same colors of extreme points of the chosen interval. [Right panel] Partial correlation plot computed by plotting the Tree Cover (%) on the x-axis against the Shapley values on the y-axis.

**
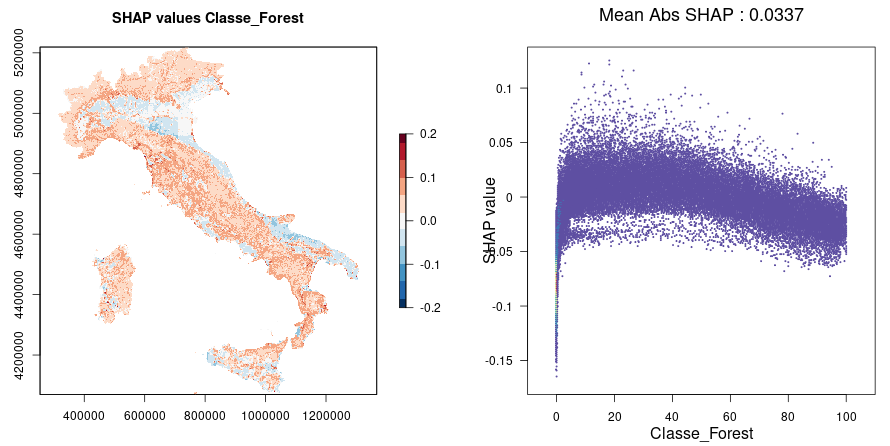
**

**Figure B.6.** [Left panel] Map of the Corine Forest Class Shapley values. SHAP values exceeding the [-0.2,0.2] range were assigned the same colors of extreme points of the chosen interval. [Right panel] Partial correlation plot computed by plotting the percentage of Forest class on the x-axis against the Shapley values on the y-axis.

**
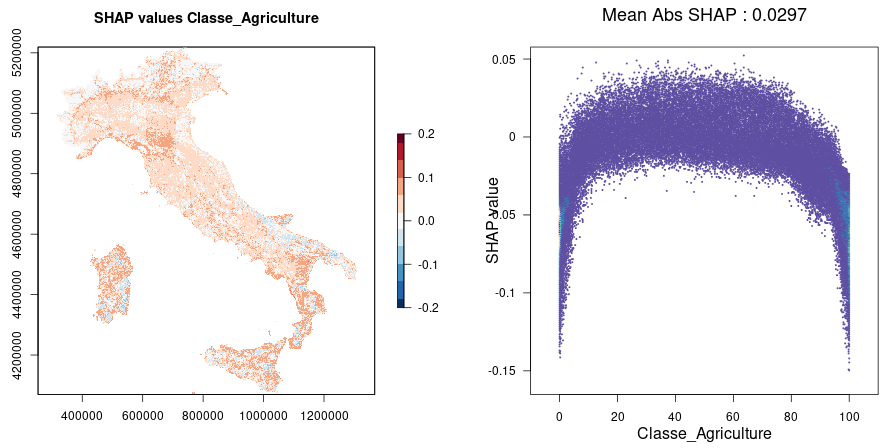
**

**Figure B.7.** [Left panel] Map of the Corine Agriculture Class Shapley values. SHAP values exceeding the [-0.2,0.2] range were assigned the same colors of extreme points of the chosen interval. [Right panel] Partial correlation plot computed by plotting the percentage of Agriculture class on the x-axis against the Shapley values on the y-axis.

**
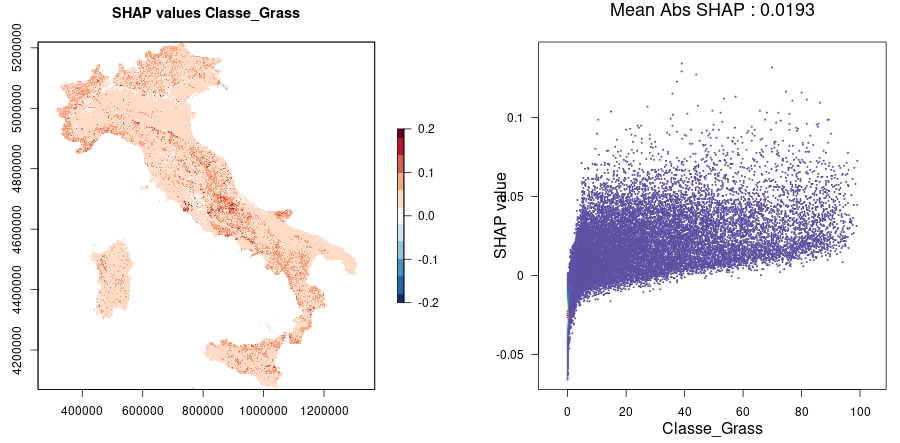
**

**Figure B.8.** [Left panel] Map of the Corine Grass Class Shapley values. SHAP values exceeding the [-0.2,0.2] range were assigned the same colors of extreme points of the chosen interval. [Right panel] Partial correlation plot computed by plotting the percentage of Grass class on the x-axis against the Shapley values on the y-axis.

**
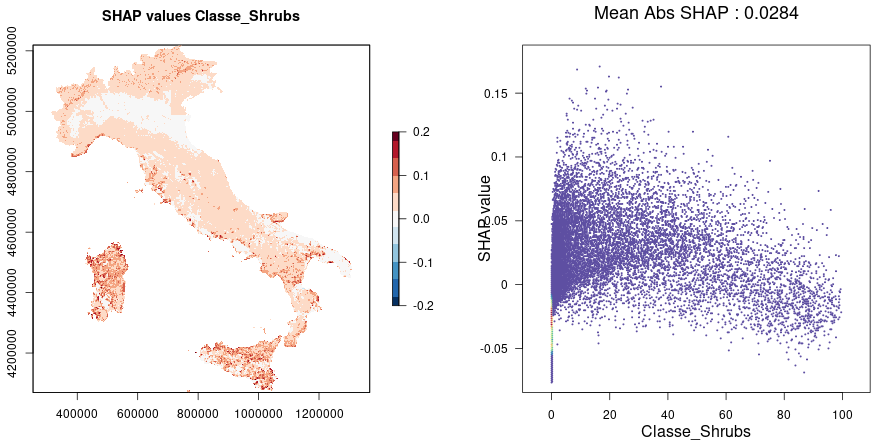
**

**Figure B.9.** [Left panel] Map of the Corine Shrubs Class Shapley values. SHAP values exceeding the [-0.2,0.2] range were assigned the same colors of extreme points of the chosen interval. [Right panel] Partial correlation plot computed by plotting the percentage of Shrubs class on the x-axis against the Shapley values on the y-axis.

**
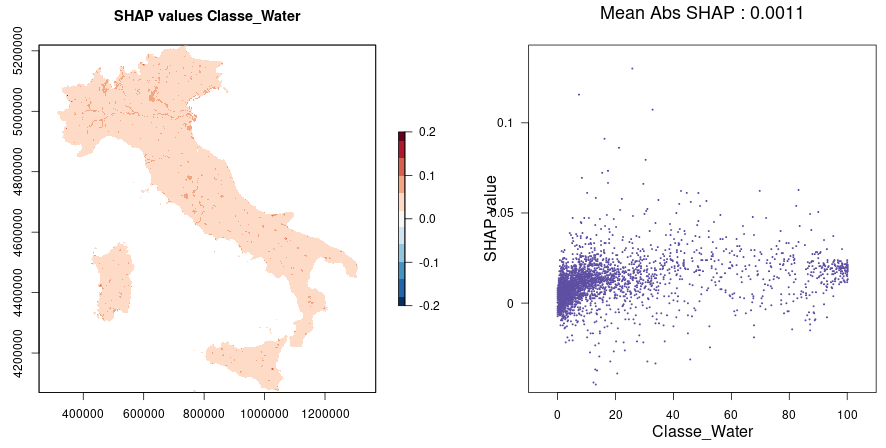
**

**Figure B.10.** [Left panel] Map of the Corine Water Class Shapley values. SHAP values exceeding the [-0.2,0.2] range were assigned the same colors of extreme points of the chosen interval. [Right panel] Partial correlation plot computed by plotting the percentage of Water class on the x-axis against the Shapley values on the y-axis.

**
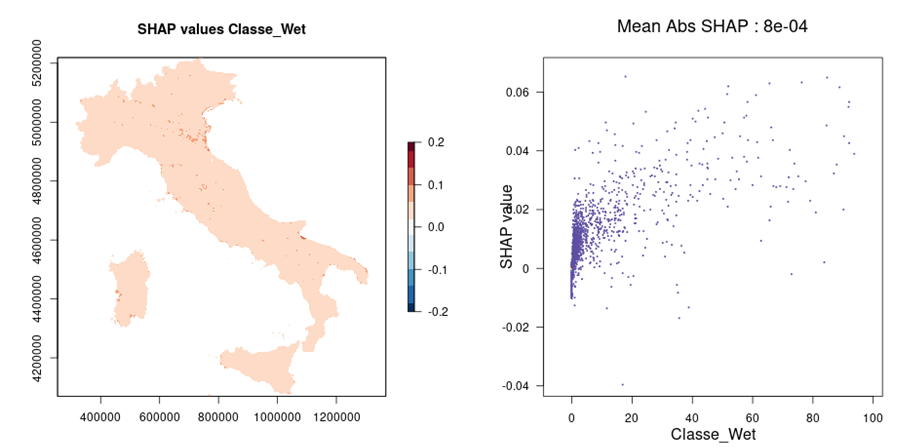
**

**Figure B.11.** [Left panel] Map of the Corine Wet Class Shapley values. SHAP values exceeding the [-0.2,0.2] range were assigned the same colors of extreme points of the chosen interval. [Right panel] Partial correlation plot computed by plotting the percentage of Wet class on the x-axis against the Shapley values on the y-axis.

**
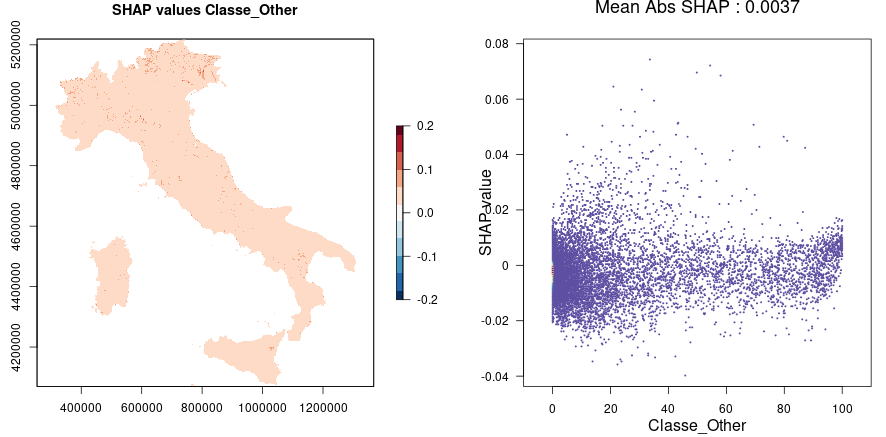
**

**Figure B.12.** [Left panel] Map of the Corine “Other” Class Shapley values. SHAP values exceeding the [-0.2,0.2] range were assigned the same colors of extreme points of the chosen interval. [Right panel] Partial correlation plot computed by plotting the percentage of “Other” class on the x-axis against the Shapley values on the y-axis.

**
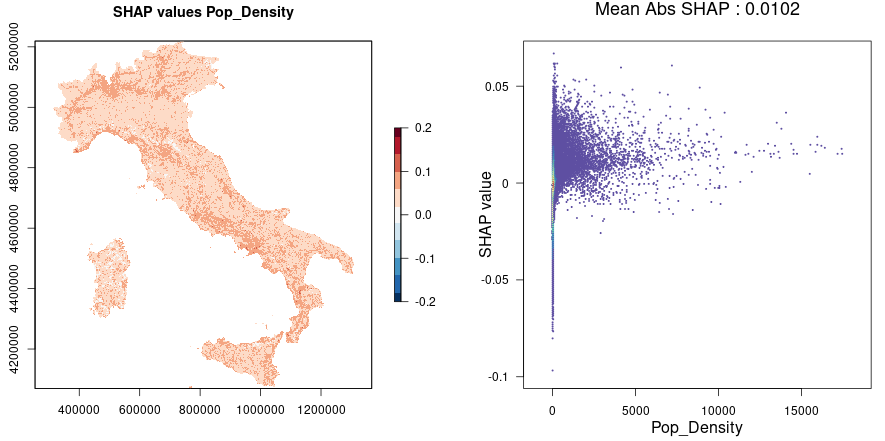
**

**Figure B.13.** [Left panel] Map of Population Density Shapley values. SHAP values exceeding the [-0.2,0.2] range were assigned the same colors of extreme points of the chosen interval. [Right panel] Partial correlation plot computed by plotting the population density values on the x-axis against the Shapley values on the y-axis.

**
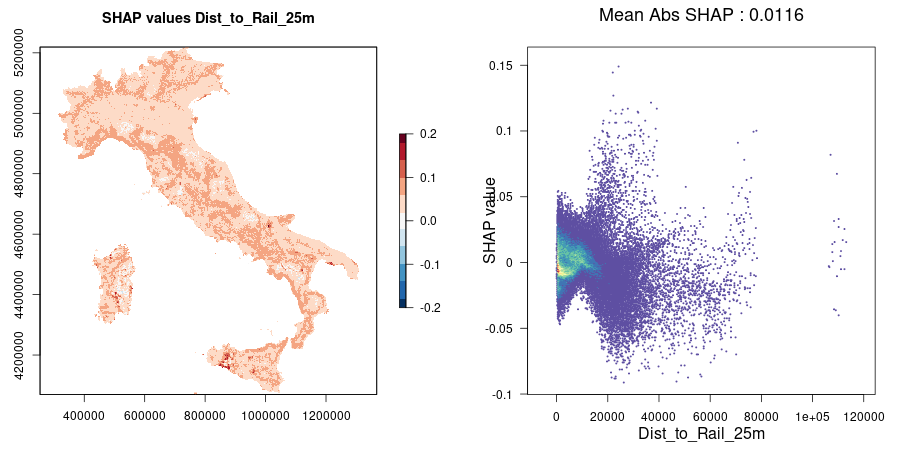
**

**Figure B.14.** [Left panel] Map of “distance to railways” Shapley values. SHAP values exceeding the [-0.2,0.2] range were assigned the same colors of extreme points of the chosen interval. [Right panel] Partial correlation plot computed by plotting distances to railways on the x-axis against the Shapley values on the y-axis.


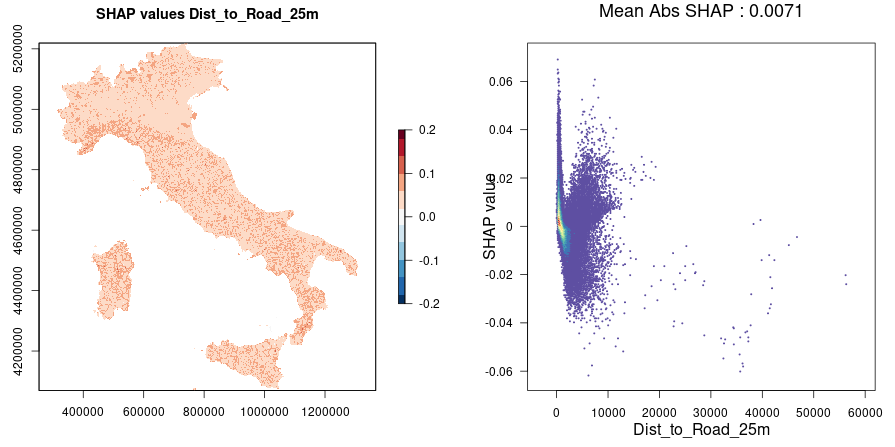


**Figure B.15.** [Left panel] Map of “distance to roads” Shapley values. SHAP values exceeding the [-0.2,0.2] range were assigned the same colors of extreme points of the chosen interval. [Right panel] Partial correlation plot computed by plotting distances to roads on the x-axis against the Shapley values on the y-axis.

**
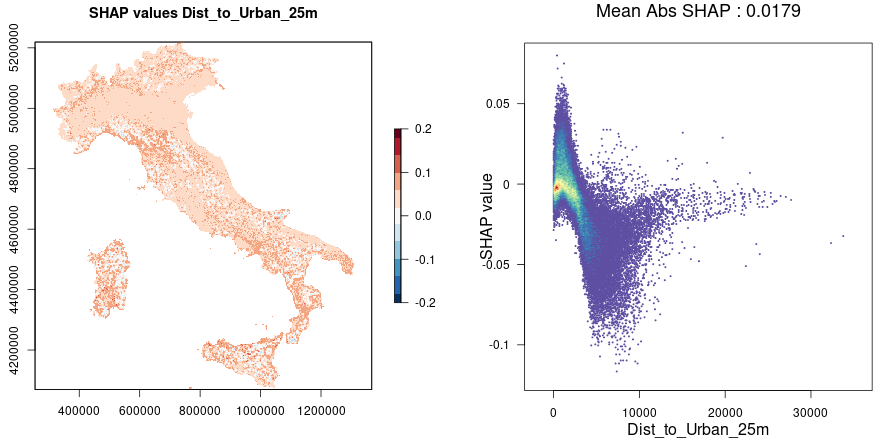
**

**Figure B.16.** [Left panel] Map of “distance to urban settlements” Shapley values. SHAP values exceeding the [-0.2,0.2] range were assigned the same colors of extreme points of the chosen interval. [Right panel] Partial correlation plot computed by plotting distances to urban settlements on the x-axis against the Shapley values on the y-axis.

**Additional Information**

All the maps provided in the paper and in the supplementary materials were created using open-source software QGIS 3.22.4 and R 4.1.2 (“raster” package v3.5.21). URL links to open-source software:

QGIS 3.22.4 -- https://blog.qgis.org/2021/10/30/qgis-3-22-bialowieza-is-released/

R 4.1.2 -- https://cran.r-project.org/bin/linux/ubuntu/fullREADME.html

“raster” package v3.5.21 -- https://cran.r-project.org/web/packages/raster/index.html
